# Legacy Effects of Canopy Disturbance on Ecosystem Functioning in Macroalgal Assemblages

**DOI:** 10.1371/journal.pone.0026986

**Published:** 2011-10-31

**Authors:** Leigh W. Tait, David R. Schiel

**Affiliations:** Marine Ecology Research Group, School of Biological Sciences, University of Canterbury, Christchurch, New Zealand; National Institute of Water & Atmospheric Research, New Zealand

## Abstract

Macroalgal assemblages are some of the most productive systems on earth and they contribute significantly to nearshore ecosystems. Globally, macroalgal assemblages are increasingly threatened by anthropogenic activities such as sedimentation, eutrophication and climate change. Despite this, very little research has considered the potential effects of canopy loss on primary productivity, although the literature is rich with evidence showing the ecological effects of canopy disturbance. In this study we used experimental removal plots of habitat-dominating algae (Order Fucales) that had been initiated several years previously to construct a chronosequence of disturbed macroalgal communities and to test if there were legacy effects of canopy loss on primary productivity. We used *in situ* photo-respirometry to test the primary productivity of algal assemblages in control and removal plots at two intertidal elevations. In the mid tidal zone assemblage, the removal plots at two sites had average primary productivity values of only 40% and 60% that of control areas after 90 months. Differences in productivity were associated with lower biomass and density of the fucoid algal canopy and lower taxa richness in the removal plots after 90 months. Low-shore plots, established three years earlier, showed that the loss of the large, dominant fucoid resulted in at least 50% less primary productivity of the algal assemblage than controls, which lasted for 90 months; other smaller fucoid species had recruited but they were far less productive. The long term reduction in primary productivity following a single episode of canopy loss of a dominant species in two tidal zones suggests that these assemblages are not very resilient to large perturbations. Decreased production output may have severe and long-lasting consequences on the surrounding communities and has the potential to alter nutrient cycling in the wider nearshore environment.

## Introduction

Macroalgae play a critical role in primary production [1] and habitat provision [2], and are commonly recognized as key species in structuring the biodiversity within communities in temperate marine systems [3], [4]. It has been widely documented that there is a current global decline in rocky shore habitat-forming macroalgal species from a wide range of stressors [5]–[7]. These species are often in high abundance and biomass, but susceptible to loss through both acute and diffuse stressors placed on coastal ecosystems such as increased coastal run-off and sedimentation [8]–[10], coastal development [11], impaired water quality [10], increased temperatures [12]–[14] and changes to the wave climate [15]. The loss of these habitat-forming species typically shifts systems that are structured in multiple dimensions (canopy, subcanopy and basal layers) to one dominated by low-lying, turf forming, filamentous or ephemeral algae [7], [16], [17]. The susceptibility of macroalgae to disturbance and their critical role in structuring communities makes them a prime candidate to test the long-term consequences of species loss on ecosystem function.

Although the long-term consequences of losing habitat-forming species, particularly fucoid algae, on community structure and composition are increasingly understood [18]–[22], there is relatively little information on the potential consequences to a critical ecosystem function, that of primary productivity. Macroalgal diversity has been shown to enhance primary productivity [23] and nitrogen uptake [24], at least in some circumstances, and the loss of these species could have significant impacts on the stability of nearshore ecosystems. Furthermore, there may be very little functional replacement of canopy forming macroalgae within intertidal assemblages [4], potentially making these assemblages vulnerable to prolonged shifts in community composition.

Chronosequences have been extensively used by ecologists to examine successional patterns where the long life span of species precludes time series observations of the entire successional sequence [25]. A chronosequence can be referred to as a mosaic of patches that have been developing for various lengths of time following a known disturbance. Such observations have been useful in formulating models of succession in terrestrial plant communities [25] and seagrass beds [26]. After a major disturbance, in certain cases, the community may not reach what is considered its ‘climax state,’ even after an extended period of time [16], [18]. Long-term shifts in community composition have the potential to drastically alter the functioning of macroalgal assemblages. While chronosequences have been used to elucidate patterns of succession, they may also be useful in examining changes in primary productivity over time.

Here we use a series of patches, disturbed experimentally at known times, and field-based photo-respirometry to test the legacy effects of the loss of canopy-forming algae on primary productivity. *In situ* photo-respirometry can measure primary productivity without altering target assemblages [27], thereby allowing them to recover along their natural successional trajectory. Understanding the direct and indirect effects of canopy loss on primary productivity over ecologically significant time-scales will allow insight into the role of macroalgal succession in the variability of primary productivity. The aim of our work, therefore, was to test the ability of macroalgal assemblages to recover to control (or pre-disturbed) levels of primary productivity and to determine the association of primary productivity with community composition. We test the null hypothesis that primary productivity of mid and low shore assemblages is unaffected by canopy removal and time since removal.

## Methods

### Chronosequence experiment, standardization and species cover

To test the long-term consequences of disturbance to dominant canopies on primary productivity, a combination of old and new removal plots were used. Canopy-forming algal species of the Order Fucales were removed from areas at two tidal heights (low and mid shore). In mid-shore algal assemblages (tidal elevation of 0.8–0.9 m), the dominant fucoid *Hormosira banksii* was removed from 3 m×3 m experimental plots in June 2002 at two sites (North Reef Moeraki 45°11′S, 170°98′E and Wairepo Reef Kaikoura, 42°25′S, 173°42′E) to test its role in ameliorating stress to the mid-shore assemblages of subcanopy algae and invertebrates [16], [21]. *Hormosira* reaches lengths of around 40 cm, with densities of hundreds of plants per m^2^ and a biomass of around 7 kg per m^2^ [4]. In the low shore zone (tidal elevation of 0–0.25 m), the large fucoid *Durvillaea antarctica* was removed from 2 m×2 m plots (n = 4) at North Reef, Moeraki and Oaro Reef, Kaikoura (42°30′S, 173°30′E) in March 2006 to examine the role of *D. antarctica* in driving community composition. *D. antarctica* adults can reach 10 m in length, densities of several plants per m^2^, and a biomass of 70 kg per m^2^. Because the very uneven limestone and conglomerate reef at Oaro Reef rendered it impossible to attach the incubation chambers effectively (see below), this site could not be used for photo-respirometry. In both mid and low intertidal assemblages, therefore, we used these plots to gauge the long-term consequences of canopy loss to primary productivity.

To gauge shorter term effects, we then added new canopy removal treatments in the mid-shore during March 2008 (Moeraki) and January 2010 (Kaikoura) and in the low-shore (Moeraki) in September 2009. New controls with *H. banksii* (mid-shore) and *D. antarctica* (low shore) canopies intact (n = 3), were also established, to produce a balanced design along with the old controls, which remained with *H. banksii* (mid-shore) and *D. antarctica* (low shore) canopies intact [16]. This chronosequence of canopy disturbance enabled us to test the trajectory of recovery of primary productivity following single episodes of major disturbance.

Incubations measuring primary productivity were done using custom-designed photo-respirometry chambers [27]. These were sealed around target assemblages immediately prior to incubations and removed after incubations to limit any long-term disturbance to the target assemblage. This allowed assemblages to be exposed to natural conditions and sampled over time with minimal impact [27]. Chambers were 25 cm in diameter, had a volume of 14.7 L, and covered 491 cm^2^ of substratum. Before attachment of chambers, a two-compound epoxy resin was used to fill in deeper cracks within the substratum so that chambers could be sealed, but care was taken not to change the reef composition such that pooling of water occurred. Before incubations were done, all visible invertebrates were removed from the assemblages to limit the effects of heterotrophic respiration. On the flat reef surfaces, the only manipulation of the reef was the drilling of holes for rawl plugs for attachment of chambers. Oxygen concentrations were sampled using a Hach LDO meter at intervals no greater than 20 minutes to avoid super-saturation of oxygen [27]. The change in dissolved oxygen over time was then converted to changes in carbon uptake using a P∶Q (photosynthetic quotient) ratio of 1∶1 and scaled up to carbon uptake per m^2^ of reef surface (g C m^−2^ h^−1^). During incubations, irradiance was measured using HOBO irradiance and temperature loggers (cross-calibrated with Li-Cor LI 192 quantum sensor) and averaged for the period of the incubation. Plots were sampled across a range of natural irradiance intensities (from 0–2100 µmol m^−2^ s^−1^), with single plots often taking multiple days to sample. Primary productivity for each treatment was then taken as the maximum productivity (P_max_) per sample area and adjusted to g C m^2^ hr^−1^; it was also standardized as productivity per dry biomass of algal material mg C gDW^−1^ h^−1^ to take account of the changing biomass per area in the experimental plots. Because treatment and control plots were re-visited throughout the years of the experiment, harvesting plots for biomass was not plausible, but dry biomass was estimated using length/mass relationships of *D. antarctica* and percent cover/mass relationships for *H. banksii*.

In addition to assessing primary productivity, the cover and diversity of species were measured at each sampling time. This was done so that primary productivity could be related to recovery and succession through time as the species composition changed. During primary productivity sampling, all macroalgal species within the areas covered by the chambers were recorded and their percentage cover estimated using a gridded quadrat. Multi-dimensional scaling plots (MDS) using PRIMER were used to visualize the variation and recovery trajectory in community structure between treatments through time. Differences between treatment plots through time were analyzed using PERMANOVA (using 9999 simulation permutations; [28]) analysis. For analysis, all replicate plots (n = 3) were plotted independently, but for graphical presentation replicate plots were averaged for each time period and only the centroid of each treatment at each sampling time was plotted. The data for percent cover of *H. banksii* and *D. antarctica* were removed from MDS analysis and PERMANOVA analysis, as is standard procedure in removal-type experiments because these distort community analyses between treatments.

### Sequence of primary productivity

One incubation chamber was set up within each of the old experimental treatments on the mid-shore. When we began the primary productivity studies, these plots were 78 (Moeraki) and 90 (Kaikoura) months old (6.5 and 7.5 years respectively). In the new removal treatments, the *H. banksii* canopy was removed from three 0.5×0.5 m areas, within which the incubation chambers were situated. Moeraki plots were sampled in spring 2008, autumn 2009 and spring 2009 giving the corresponding chronosequence of 0, 6, 12, 78, 84 and 90 months after canopy disturbance. Kaikoura plots were sampled in austral summer 2010 and winter 2010, giving the corresponding chronosequence of 0, 6, 90 and 96 months after canopy disturbance.

Similarly on the low shore, one incubation chamber was set up within each of the old experimental removal and control plots. These plots were 30 months old when we began the primary productivity studies. In the new removal plots, *D. antarctica* adults were removed from three 0.5×0.5 m areas, within which the incubation chambers were situated. Incubations testing primary productivity in all treatments were done during spring 2009 and autumn 2010. This gave a chronosequence of 0, 6, 30 and 36 months after canopy disturbance. Because of the large sizes that *D. antarctica* plants can reach, controls were set up around moderate sized plants (no taller than 50–60 cm) because larger ones would not fit inside chambers.

## Results

### Sequence of primary productivity in mid-shore communities

**Figure 1:**
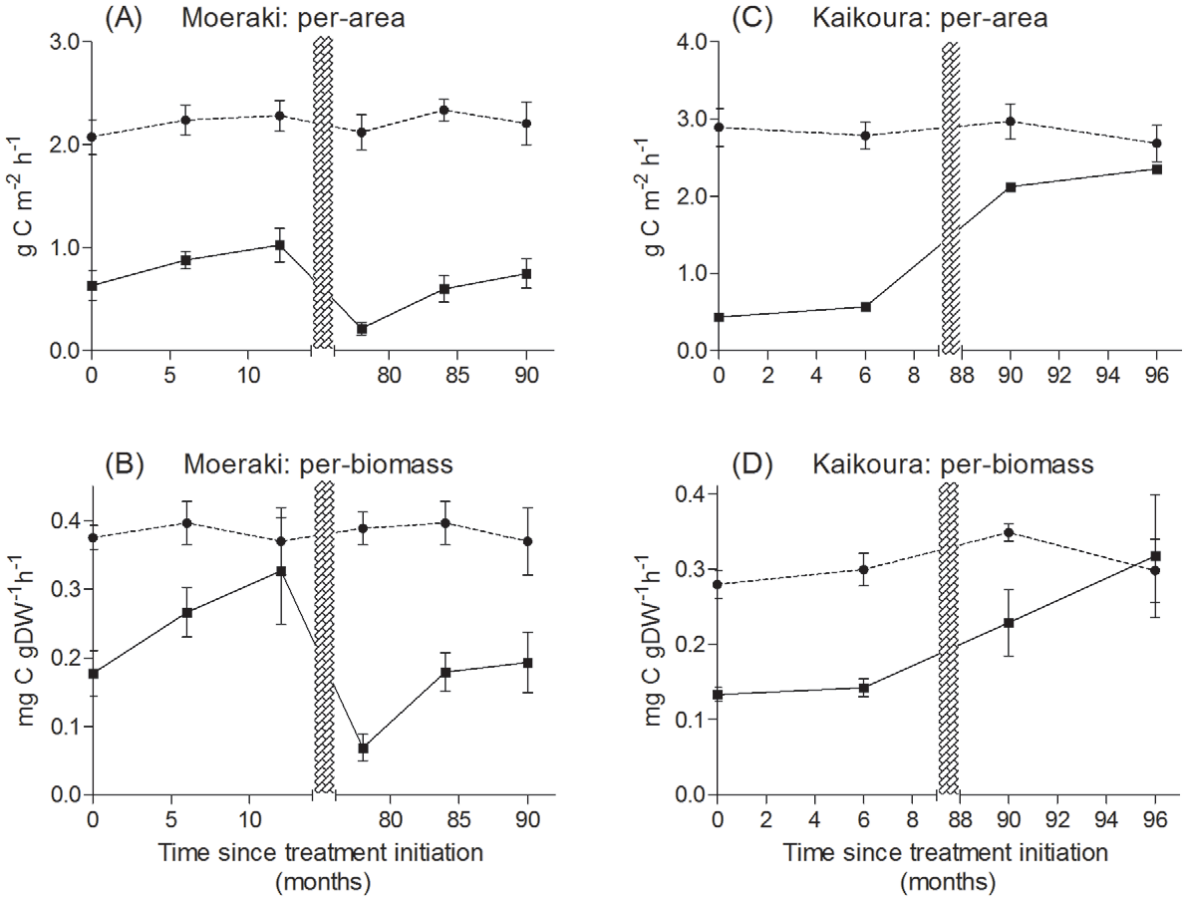
Primary productivity (±SE) in mid intertidal zone control (dashed lines) and canopy (*Hormosira banksii*) removal (solid lines) plots in two sites through time, standardized on a per-area basis (Moeraki (A) and Kaikoura (C)) and per-biomass basis (Moeraki (B) and Kaikoura (D)). Shaded bar indicates the change from new to old treatments (see text).

**Figure 2:**
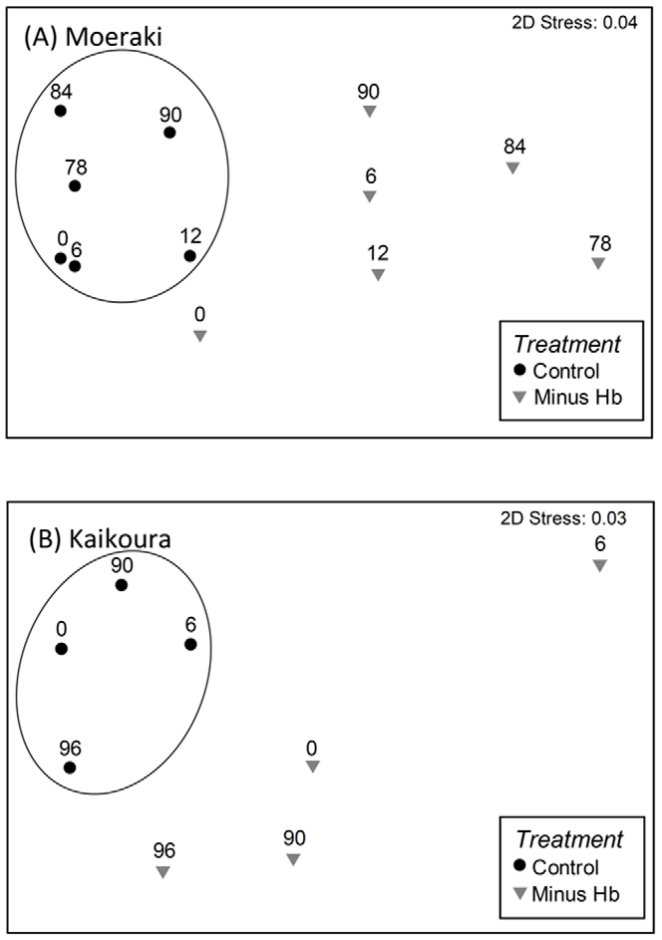
MDS plots of changing community composition in mid intertidal zone control (intact assemblages), and canopy (*Hormosira banksii*) removal plots through time at Moeraki (A) and Kaikoura (B). Each point represents the centroid of three replicate plots. Numbers above symbols indicate the number of months since the initiation of treatments.

The loss of the *H. banksii* canopy had a major impact on the productivity of the mid- shore assemblages of both sites (Fig. 1). The immediate effect was a reduction of primary productivity from 2.1 to 0.6 g C m^−2^ h^−1^ at Moeraki (Fig. 1A) and 2.9 to 0.4 g C m^−2^ h^−1^ at Kaikoura (Fig. 1C). Primary productivity results were similar when standardized to biomass, with a c. 50% reduction in removal treatments at both sites (Figs. 1B and D). In all cases, the lower values in the *Hormosira* removal plots represented the productivity of the remaining understory algal assemblage and indicate that both on a per-area and per-biomass basis, the loss of the canopy fucoid resulted in a significant loss of primary productivity. The differences between treatments and sites persisted throughout the first year at Moeraki and at least for 6 months at Kaikoura (per-area Treatment×Site interaction at 6 months: F*_8,11_* = 10.22, p = 0.013). The long-term plots, however, showed increasing differences between the two sites. After 90 months, primary productivity in removal plots at Moeraki did not recover beyond initial levels, but those at Kaikoura had recovered much of their primary productivity (per-area Treatment×Site interaction: F*_8,11_* = 5.57, p = 0.046). We were able to do an additional set of incubations at Kaikoura. This showed that after 8 years (96 months) there was finally a convergence of treatment and control plots in primary productivity (per-area: F*_1,4_* = 1.96, p = 0.23). Multi-dimensional scaling plots illustrated the large variation in assemblage structure in the removal treatments over time at both sites (Fig. 2). Treatments diverged greatly after six months, as the remaining algal assemblages in removal plots responded to the loss of the canopy and increasing stress in the mid tidal zone. PERMANOVA analysis of community composition at Moeraki showed a significant difference between treatments (F*_1,24_* = 16.8, p<0.01), times (F*_5,24_* = 7.6, p<0.01) and a significant interaction (treatment×time, F*_5,24_* = 2.1, p<0.05). The poor recovery at this site was indicated by the continued divergence of the community, even after 90 months (Fig. 2A). Furthermore, although *H. banksii* cover had recovered somewhat, it was still lower than controls (95% cover in controls, 78% cover in removals). The control plots had more fucoid algae, especially *Cystophora* spp, and branching red algae (*Lophothamnion hirtum*). Removal plots had more turfing coralline algae, especially a greater cover of *C. officinalis* and *Jania micrathrodia*, and more ephemeral species. The cover of corallines varied considerably over time due to burn-off during the summer months. After 6 months, *H. banksii* had started recruiting into removal plots but their cover and biomass were far less than in controls. Ninety months after initial removal, there was a similar species composition in control and removal plots, with very similar cover of *Corallina officinalis* and encrusting corallines. However, *H. banksii* density and biomass remained less than in controls [29], which translated into the large differences seen in primary productivity.

At Kaikoura, the algal community composition of removal plots was very different to controls as early as 6 months after canopy removal (Fig. 2B). This was caused by the die-back of corallines and the abundance of ephemeral, disturbance-oriented species such as *Ulva, Colpomenia* and *Adenocystis*. PERMANOVA analysis showed a significant effect of treatment (F*_1,16_* = 6.5, p<0.05) and time (F*_3,16_* = 2.5, p<0.01), but no interaction. Throughout the time series, there were generally fewer fucoids, more corallines and more ephemerals in removal plots relative to controls. After 96 months, removal treatments were similar to controls (Fig. 2 B). The *H. banksii* canopy recovery was much quicker at Kaikoura than at Moeraki, and there was around a 90% canopy cover of recruits after approximately 2 years, which grew into full cover of large plants. At both sites, however, species richness and the abundance of mid-canopy fucoids were still lower in removal plots than controls after 90 months at both Kaikoura and Moeraki [29].

### Sequence of primary productivity in low shore communities

**Figure 3:**
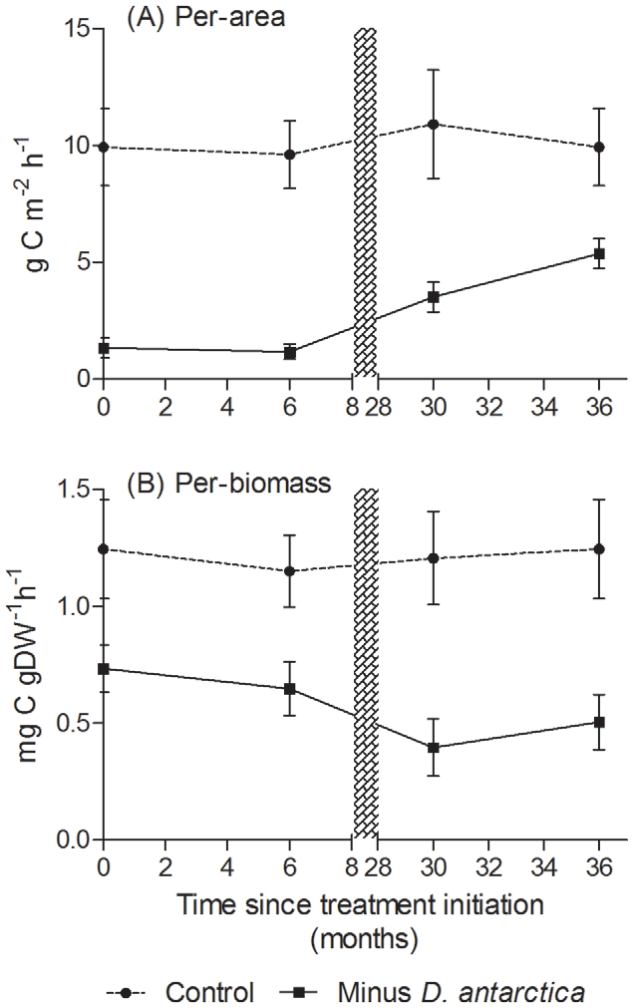
Primary productivity (±SE) in low intertidal zone control (dashed lines) and canopy (*Durvillaea antarctica*) removal (solid lines) plots at Moeraki, standardized on a per-area basis (A) and per-biomass basis (B). Shaded bar indicates the change from new to old treatments (see text).

Removal of the *Durvillaea antarctica* canopy had a large initial impact on per-area productivity (F*_1,4_* = 17.2, p = 0.014), which persisted at the same level for at least 6 months (F*_1,4_* = 37.0, p = 0.004; Fig. 3A). The controls had an average of around 10 g C m^−2^ h^−1^ compared to the removals at about 1.3 g C m^−2^ h^−1^. The older removal treatments showed that recovery of plots had begun by 30 months after canopy removal but significant differences remained after 36 months (F*_1,4_* = 9.99, p = 0.034), with controls at an average of 10.4 g C m^−2^ h^−1^ and removal plots at 5.4 g C m^−2^ h^−1^. These levels of primary productivity in *Durvillaea* rival that of other large macroalgae such as *Macrocystis pyrifera* [30]. Although less pronounced, there was also a significant effect of *D. antarctica* removal on a per dry biomass basis (F*_1,4_* = 8.24, p = 0.041, Fig. 3B). Between 0–6 months after canopy removal, per-biomass productivity in removal plots was approximately 50% of the control treatment (compared to c. 15% when standardized by reef area), which indicates the large per-capita contribution that *Durvillaea* makes to productivity. On both a per-area and per-biomass basis, therefore, its loss greatly affected productivity.

**Figure 4:**
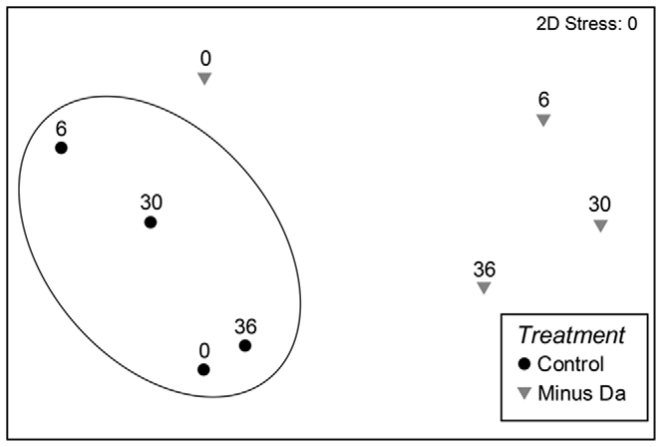
MDS plots of changing community composition in low intertidal zone control (intact assemblages), and canopy (*Durvillaea antarctica*) removal in plots through time at Moeraki. Each point represents the centroid of three replicate plots. Numbers above symbols indicate the number of months since the initiation of treatments.

MDS plots of community composition show an obvious separation between treatments in multi-dimensional space, which increased through 6 and 30 months and began converging by 36 months (Fig. 4). PERMANOVA analysis showed there were significant differences between treatments (F*_1,16_* = 6.0, p<0.05) and time (F*_3,16_* = 1.8, p<0.05). These differences involved a) the shifting proportions of assemblages between *D. antarctica* and *Corallina officinalis* in control plots, and b) expanded cover of corallines, incursions of the invasive kelp *Undaria pinnatifida*, and recruitment of the low-shore fucoid *Cystophora torulosa* in removal plots. Removal plots had a 30% increase in the cover of coralline turf (*Haliptilon roseum* and *C. officinalis*) in the first 6 months, but an overall decline in algal diversity. After 30 months, the removal plots had a large variation in species diversity and cover, with plots dominated by *C. torulosa*, *U. pinnatifida*, *C. officinialis* and the subtidal fucoid *Xiphophora gladiata*, but with increasing cover of juvenile *D. antarctica* by 36 months. In control plots, the canopy cover of *D. antarctica* was almost 100% at all sampling intervals, although there was some natural variation in the subcanopy composition.

## Discussion

The loss or severe reduction of canopy-forming algae in the nearshore zone has produced changes in communities worldwide, usually as a result of multiple stressors [31]. Where fucoid algae are involved, the trajectory of recovery is often slow, sometimes over decades [18]–[22]. The consequences of these changes on one of the most important ecosystem functions, that of primary productivity, are generally not known. Here, however, we show that reduced productivity occurs for several years in both the mid and low intertidal assemblages, primarily because of the slow pace of recovery of canopy species [29] and the advent of lower-lying turfs and fleshy algae.

Community changes following fucoid canopy impacts are complex and varied among different regions. On British shores, for example, loss of a dominant may allow other fucoids to establish, involve grazer dynamics and eventual slow re-establishment of the original dominant species [32]. In many areas where stressors such as coastal sedimentation persist, there is an increased abundance of low-lying turf species [5]–[7]. Although it was not tested in this study, higher amounts of sedimentation, associated with greater coralline cover [29], and an increase in the wave climate [15] at the southern site of our study may have slowed recruitment of *Hormosira banksii* compared to the northern site of Kaikoura. Along the shores of southern New Zealand and southeastern Australia, the loss or reduction of the mid intertidal dominant, *Hormosira banksii*, produces variable responses. For example, Underwood [18], [33] showed that a severe storm removed large patches of this species and that its recovery over several years was affected by both direct and indirect effects of grazers. Bellgrove et al [34] speculated for shores further south in Australia that patches of *Hormosira* and coralline turfs represented alternate states of intertidal communities. Schiel [15], however, showed that over 17 years, *Hormosira* canopies came and went in response to storms and wave damage, and that canopy losses in the mid intertidal zone triggered a series of cascading losses of assemblage structure, as newly exposed understory species became desiccated and died off. These were often replaced for several years by coralline turfs and seasonally blooming ephemeral species before eventual recovery of the *Hormosira* canopy. It was experimentally shown, however, that even after 8 years, there were still differences in biomass and diversity between canopy-impacted areas and controls [29]. Our study shows the resulting compromised primary productivity over several years.

In both the mid and low intertidal zones, the removal of the dominant fucoid canopy immediately resulted in a large fall in primary productivity. This is hardly surprising as both the dominant biomass and much of the areal cover of algae were lost. Biomass has often been used as a surrogate for rates of primary productivity in macroalgae [30]. Our study showed that biomass was a key determinant of overall per-area primary productivity, but when productivity was expressed per-biomass there was still significantly lower productivity in removal treatments, indicating that biomass alone does not entirely explain the loss of productivity and that certain taxa are disproportionately important. In particular, the dynamics of light use by the species that dominated canopy removal areas play a key role in this response. For example, on both a per-area and per-biomass basis, corallines are some of the least productive algae. Their calcium carbonate structure makes them heavy relative to their photosynthetic capability [35]. In contrast to corallines, the ephemeral species that bloomed in removal areas had high productivity on a per-biomass basis, but were patchy and did not have great productivity on a per-area basis. Species such as sea lettuce (*Ulva* spp), for example, in a full light environment can have productivity as high as 10 mg C gDW^−1^ h^−1^ [35], compared to corallines at 0.3 mg C gDW^−1^ h^−1^ [27]. Similar high levels of per-biomass productivity occur for other ephemeral algae [35]. In intact assemblages with a full fucoid canopy, these species remain in the understory, along with several species of low-shore fucoids, and contribute to the overall assemblage primary productivity at high light intensities [36]. However, when fully exposed to a high light environment following canopy removal, they can undergo photo-inhibition [37], and often desiccate and bleach. In contrast to the loss of smaller fucoids higher up on the shore, the loss of the much larger “bull kelp” *Durvillaea antarctica* on the low shore removes the abrasion of the substratum by fronds [38], and results in elongation of corallines and the recruitment of lower biomass species with far less areal productivity. Even an incursion by the invasive annual kelp *Undaria* did not compensate in primary productivity for the loss of *Durvillaea*.

If the results seen in our study apply to assemblages elsewhere, where the loss of dominant algal canopies has led to long-term changes in communities, then there are potentially large consequences to benthic primary productivity for algal communities worldwide. Given the susceptibility of large, canopy-forming macroalgae to disturbance [8]–[14], understanding how their loss affects important ecosystem functions such as primary productivity is essential. Major alterations to carbon fixation through primary productivity have the potential to alter the functioning of ecosystems and the functioning of global biogeochemical cycles [39]. Decreased carbon output could have far-reaching consequences, with stable isotope signatures indicating the importance of macroalgae well beyond the nearshore environment [40], [41]. The decline of dominant canopy-forming macroalgae worldwide [5] has the potential, therefore, to alter carbon fixation of nearshore ecosystems, with consequences reaching far up the food chain.
